# Long-Term Hypoparathyroidism and Hypophosphatemia in Dialysis Patients

**DOI:** 10.1177/2324709614527258

**Published:** 2014-05-06

**Authors:** Linda Shavit, Meyer Lifschitz, Itzchak Slotki

**Affiliations:** 1 Shaare Zedek Medical Center, Jerusalem, Israel; 2 University of Texas Health Science Center at San Antonio, Texas

**Keywords:** dialysis patients, hypoparathyroidism, hypophosphatemia

## Abstract

*Background and Objectives*. Hypoparathyroidism in patients with functioning kidneys leads to hyperphosphatemia. This article reviews data suggesting that hypoparathyroidism in patients on dialysis leads to hypophosphatemia. *Design*. Clinical data of the following were reviewed: (*a*) a patient with hypoparathyroidism before and during chronic dialysis; (*b*) patients on dialysis with surgically created hypoparathyroidism; (*c*) dialysis patients being treated with Cinacalcet, a calcium-sensing receptor agonist that lowers parathyroid hormone (PTH) levels; and (*d*) dialysis patients being treated with Velcalcetide, a new calcium-sensing receptor agonist that also lowers PTH. *Results*. In the patient presented in this study, in patients with surgically created hypoparathyroidism, and those receiving Cinacalcet or Velcalcetide, a fall in PTH was associated with hypophosphatemia or a fall in serum phosphorus. *Conclusion*. In patients on dialysis, hypoparathyroidism may lead to hypophosphatemia.

## Introduction

In patients with chronic renal failure, including those on chronic dialysis (end-stage renal disease [ESRD]), both hyperparathyroidism and hyperphosphatemia are regularly present. The number of patients with chronic renal failure and hypoparathyroidism is few,^1-4^ and the results of hypoparathyroidism on serum phosphorus (Pi) in these patients have infrequently been reported in the literature. This report includes the following: (*a*) clinical information of a patient who, when originally seen, had normal serum creatinine and was hypoparathyroid and hyperphosphatemic, and later when he was a chronic dialysis patient for 11 years his hypoparathyroidism was associated with hypophosphatemia; (*b*) reviews the published results of selected dialysis patients with surgically induced hypoparathyroidism with hypophosphatemia; (*c*) summarizes published results with Cinacalcet, which has been used to lower parathyroid hormone (PTH) in ESRD patients on dialysis, and which regularly lowers serum Pi; and (*d*) reviews published results with Velcalcetide, a new drug that also lowers PTH in ESRD patients on dialysis and which is also associated with a low serum Pi.

## Methods

For the case report, hospital and dialysis unit records were reviewed and summarized. Where multiple laboratory values were available, the results were averaged for inclusion in Table 1. Additional cases of patients with hypoparathyroidism on dialysis were found by searching PubMed, Uptodate, and Google Scholar. Similar search efforts were used to find case series using Cinacalcet in dialysis patients. Only reports in which serum Pi was reported were included. A single report exists using Velcalcetide in dialysis patients.

**Table 1.** Laboratory Values Including Serum Calcium, Phosphorus, and PTH of the Patient^a^.

	Case 1								
	1994	2002	2003	2004	2006	2007	2008	2009	2012
PTH, pg/mL	36	15	15	19	6	15.7	11	15	14.4
Ca, mg/dL	4.8	8.4	7.4	6.4	7.9	8.2	8.7	8.1	8.4
Pi, mg/dL	5.4	1.5	1	1	1	0.9	1.1	1.4	1.9
Albumin, g/dL	4.1	3.9	4	4.5	3.7	3.8	4.1	3.8	3.5
Creatinine, mg/dL	1.2	5.5	6	8.6	8.53	6.58	5.69	6.95	4.9
Total cholesterol, mg/dL	170	161	123	128	113	134	124	124	131
Triglyceride, mg/dL		98	94	68	61	83	70	57	66
Hemoglobin, g/dL	11	—	11.5	—	11.8	11.9	12.9	10.8	11

Abbreviation: PTH, parathyroid hormone.

a Normal range for the laboratory values: Ca, 8.0-10.5 mg/dL; Pi, 2.5-5.0 mg/dL; and PTH, 16.0-87.0 pg/mL. The data from 1994 were 8 years before he started chronic hemodialysis. From 2002 to 2013, he was on chronic hemodialysis for 4 hours 3 times per week. The patient had low PTH, Ca, and Pi and did not undergo surgical parathyroidectomy. Other nutritional parameters indicate adequate nutrition during follow-up.

### Results in Patient

The patient is a 58-year-old man whose medical history was uneventful until 1990 when he was hospitalized following acetic acid ingestion with injury to both his esophagus and respiratory system. He was treated with a feeding gastrostomy and tracheostomy and recovered. In 1992, he developed a jejunal perforation that was repaired with a gastrojejunostomy and had a segment of his colon transplanted to replace his injured esophagus. In 1994, hypocalcemia and hyperphosphatemia with a relatively low PTH (Table 1) was noted. His TSH levels were normal, and a search for an immunologic basis for his hypoparathyroidism was unsuccessful (negative anti-thyroglobulin, anti-microsomal, anti-smooth muscle, anti-mitochondrial and anti-parietal cell antibodies). In 1995, he developed a bowel obstruction, and in 1998, he was given the diagnosis of chronic obstructive pulmonary disease. In 2001, he was found to have bilateral small kidneys with hypertension and proteinuria and was started on oral calcium carbonate and active vitamin D_3_ (Alphacalcidol [1-alpha-OH D3] Teva) for a serum calcium (Ca) of 7.1 mg% and Pi of 1.0 mg%. He was started on regular outpatient hemodialysis, and from January 2002 to the present his routine blood chemistries demonstrate hypocalcemia, hypophosphatemia, and very low PTH levels (Table 1). The patient received outpatient hemodialysis using a Fresenius machine (Fresenius Medical Care, Bad Homburg, Germany). The dialysis sessions were 4 hours each 3 times per week with polysulfone hollow fiber dialysers (Fresenius Medical Care), and bicarbonate-based dialysate was used. Dialysate Ca was 1.5 mmol/L. The blood flow rate varied between 250 and 300 mL/min and was recorded by the dialysis machine flowmeter. The dialysate flow was kept constant at 500 mL/min. Dialysis adequacy was assessed by Kt/v. This was regularly measured every 3 months and was in the normal range for chronic dialysis patients (1.24-1.41).

For the last 11 years he has been regularly treated with hemodialysis using a left forearm fistula. He has no specific symptoms related to his hypophosphatemia such as muscle weakness, hemolytic anemia, central nervous system or pulmonary symptoms. His previous chronic obstructive pulmonary disease is presently asymptomatic.

Physical examination reveals a slim man with a left forearm fistula and abdominal and chest scars from his jejunal colon-esophagus surgery. His muscle strength is good, and he has no bone symptoms. Much of the last 11 years he has been given active vitamin D_3_ without Pi binders. Serum 25-OH vitamin D was 181 nmol/L (normal range = 75-250 nmol/L) in 2012. X-rays including his chest, arms, and pelvis in 1994 and 2012 demonstrated grossly normal bones. His serum albumin, creatinine, cholesterol, triglycerides, and hemoglobin are within the range of most chronic dialysis patients, suggesting that he does not have generalized malabsorption (Table 1). However, this patient might have a defect in intestinal Pi absorption, perhaps due to his toxic ingestion and/or his jejunal perforation.

### Published Reports of Dialysis Patients With Surgically Induced Hypoparathyroidism and Hypophosphatemia

Although surgical treatment of secondary hyperparathyroidism in dialysis has been used for many years, few such patients develop hypoparathyroidism. Of those that have been reported in the literature^1-3^ with hypoparathyroidism, including a report from our group,^4^ hypophosphatemia is regularly found. In several cases,^2-4^ hypophosphatemia persisted for more than a year.

### Published Reports of Dialysis Patients With Cinacalcet-Induced Hypoparathyroidism and Hypophosphatemia

For the past 13 years, there has been a rapid increase in the use of Cinacalcet, a drug that increases the sensitivity of the Ca-sensing receptor to extracellular Ca, to help control the hyperparathyroidism in chronic renal failure patients on dialysis. As shown in Table 2,^5-19^ use of Cinacalcet in this setting leads to a considerable decrease in measured PTH and a consistent, but smaller fall in serum Ca and Pi. Although data in Table 2 indicate a fall in serum Pi in each study, this change was not always statistically significant. Recently it has been reported that the use of this same drug in patients with stage 3 to 4 kidney disease is associated with an increase in serum Pi^20^ and a decrease in urinary Pi excretion.

**Table 2.** Effect of Cinacalcet in Dialysis Patients.

Reference	Dose of Cinacalcet (mg/day)	Duration of Study (Weeks)	PTH (Changes in %)	Serum Calcium (Changes in %)	Serum Phosphorus (Changes in %)	Additional Information
Goodman et al^5^	25-100	1	−55	−7	−25	a
Lindberg et al^6^	20-50	18	−22	−5	−8	a
Quarles et al^7^	100	18	−33	−5	−3	a, b
Block et al^8^	30-180	26	−43	−7	−8	
Lindberg et al^9^	30-180	10	−40	−7	−7	
Moe et al^10^	30-180	26	−57	−10	−7	a, c
Chertow et al^11^	30-180	16	−1.8	−9.7	−11.1	
Sterrett et al^12^	30-180	52	−48	−6.5	−3.6	
Lazar et al^13^	30-180	52	−30	−8.1	−10.1	
Arenas et al^14^	30-120	36	−70	−13.1	−10.4	b
Fishbane et al^15^	30-180	33	−47	−7.1	−1.2	b
Messa et al^16^	30-180	23	−46	−7	−5	
Fukagawa et al^17^	25-100	14	−54	−8.1	−10.2	
Sprague et al^18^	30-180	180	−53	−2.6	−10.5	
Raggi et al^19^	30-180	52	−32	5.2	−17.2	c

a Discussed the possibility that the decrease in serum phosphorus could be due to hungry bone syndrome.

b The decrease in serum phosphorus was not significant.

c The changes were calculated from data presented in graphic form in the reference.

### Published Report of Dialysis Patients With Velcalcetide-Induced Decrease in PTH Associated With a Decrease in Pi

Velcalcetide is a new calcimemitic that acts directly on the calcium-sensing receptor independent of calcium.^21^ In dialysis patients, single-dose administration after dialysis leads to a fall in PTH, Ca, and Pi relative to controls.

## Discussion

In patients with ESRD on chronic dialysis, there are multiple changes in the regulatory systems of Ca and Pi that are presently known. Secondary hyperparathyroidism is routinely found as is hyperphosphatemia and a lower serum Ca and 1,25-D3. FGF-23 is increased. Renal excretion of Pi is not an important consideration since ESRD patients make little or no urine. Lack of renal Pi excretion is one of the factors leading to secondary hyperparathyroidism in ESRD. Until very recently these alterations in serum chemistries and hormones were managed with oral Pi binders to diminish gastrointestinal Pi absorption and administration of an active form of vitamin D such as 1-alpha-hydroxy-D3. Although effective, it has been difficult to control the PTH with this regimen, although it does frequently decrease. In addition, hypercalcemia from excess vitamin D and hyperphosphatemia are still frequently found in ESRD patients. Thus, the development of Cinacalcet, which regularly lowers PTH, Ca, and Pi in these patients, has been well received. The effect of Cinacalcet to lower Pi in this setting has been regularly commented upon (see Table 2), but to date no studies have been published to differentiate between “hungry bone disease” and decreased intestinal Pi absorption as a mechanism for this. In fact, in some studies this change was not statistically significant.

Our patient may be the only patient in the literature with hypoparathyroidism both before and during chronic hemodialysis. This patient has been on regular hemodialysis for over 11 years, but in contrast to what is referred to above as the standard condition of such patients, including the other patients in his dialysis unit, where high PTH and Pi are expected, he regularly has very low Pi and PTH levels, the latter of which may be below the level the assay can measure. His low PTH could be a consequence of his esophagus-colon transplant surgery, that is, his parathyroids may have been injured during the surgery or their blood supply compromised leading to hypoparathyroidism. Alternatively, his low Pi may inhibit PTH synthesis as has been shown in rats.^22^ Hypocalcemia, hyperphosphatemia, and a relatively low PTH were noted years before he started on dialysis, thus indicating that he had hypoparathyroidism when his kidney function was still relatively normal (Table 1). When he first started on dialysis in 2002, he was receiving oral calcium carbonate and alpha-D3, and his serum Ca was 7.1 and serum Pi 1.0. His first PTH level on dialysis was low at 15.2. For the next 11 years he regularly had low serum Ca, Pi, and PTH (Table 1). Could his low Pi be due to malabsorption of Pi? Pi absorption is mainly found in the duodenum and jejunum. He may well have injured his duodenum and jejunum with his original acetic acid ingestion, and his subsequent jejunal perforation and its surgical treatment may have limited his intestinal capacity for Pi absorption. The transporters known to play a role in Pi absorption in the intestine have been described and the effect of PTH and vitamin D on these transporters have been described in animal models.^23^

Against this suggestion are the observations that, by other parameters, he does not have malabsorption and is not malnourished. Could he have an ongoing case of “hungry bone syndrome”? Although he was never shown to have hyperparathyroidism, perhaps his intestinal Pi, which is absorbed, is regularly deposited in his bones, which appear normal on X-ray. An alternative possibility is that his hypoparathyroidism, in some manner, inhibits intestinal Pi absorption.

Hungry bone syndrome refers to the phenomenon in which serum Ca and Pi fall following parathyroidectomy^24,25^ in patients with previous hyperparathyroidism. It is thought that following parathyroidectomy there is movement of Ca and Pi into bone. This is usually a transient condition, although it has been described as lasting for several months. If this is the explanation for the decrease in Pi with Cinacalcet, in patients following parathyroidectomy and in our patient, it would seem that it can continue for some years. Lack of a tissue diagnosis (intestinal or parathyroid biopsy) makes the final understanding of the cause for the low PTH and Pi in this patient speculative.

Hypoparathyroidism in ESRD is not regularly discussed in the literature because hyperparathyroidism rather than hypoparathyroidism is regularly found in ESRD. A review of blood PTH levels in more than 8000 Japanese patients on dialysis, performed before Cinacalcet became available, identified some patients with lower than expected PTH levels, but most other clinical parameters were not reported.^1^

Several reports of patients with ESRD following parathyroid surgery induced hypoparathyroidism exist, and in these patients hypophosphatemia has been reported as mainly due to hungry bone syndrome.^2-4^ Of course, transient hypoparathyroidism is seen in those patients who received active vitamin D and become sufficiently hypercalcemic, but this is usually quite transient and hyperphosphatemia is usually found in this setting, presumably due to the effect of vitamin D to increase intestinal Ca and Pi absorption. Now with Cinacalcet becoming widely available, relative hypoparathyroidism in dialysis patients may become more prevalent. Velcalcetide, although acting on the calcium-sensing receptor in a manner different from Cinacalcet, also has similar clinical effects.

Low PTH in dialysis patients is also associated with adynamic bone disease, but this is usually associated with excessive vitamin D administration and elevated Ca and Pi in contrast to the case of our patient discussed here.

**Figure 1.**
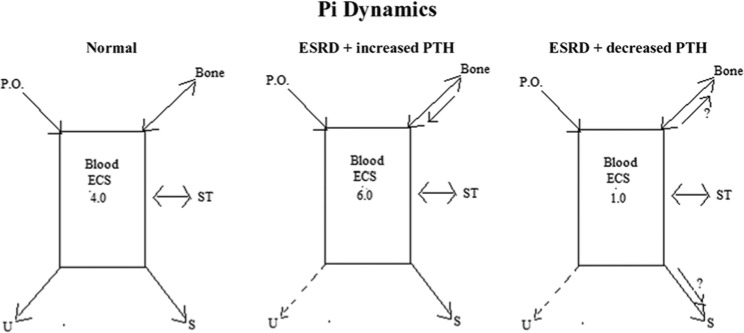
Phosphorus dynamics in 3 clinical states—normal, end-stage renal disease (ESRD) with elevated parathyroid hormone (PTH), and ESRD with decreased PTH. P.O. is oral intake; Blood ECS (extracellular space) indicates serum Pi levels; U indicates urinary excretion; S indicates stool excretion; and ST indicates soft tissues. In the Normal case, Pi is taken in with the diet, moves into and out from bones and ST, and is excreted in the urine and stool. Serum Pi is 4 mg/dL. In ESRD with increased PTH, changes include more movement out of bone and virtually no urinary excretion. Serum Pi is 6 mg/dL. In ESRD with decreased PTH, the possible increased movement of Pi into bone and increased Pi excretion is stool is indicated. Serum Pi is 1.0 mg/dL. The values for Pi are chosen as examples from data in Table 1.

The process by which low PTH leads to a fall in serum Pi in patients on dialysis is worthy of consideration. It is unlikely that an effect on renal excretion of Pi into the urine is important, since these patients make very little urine. The most obvious possibility is that in previous hyperparathyroid patients with increased bone resorption as a consequence of increased PTH, when PTH falls, bone resorption also falls, leading to less Ca and Pi coming out of bones. In some cases even net movement of Ca and Pi into bone occurs. These changes are depicted in Figure 1. Although there are no objective data in humans to indicate that low PTH or Cinacalcet diminishes intestinal Pi absorption independent of an effect on active vitamin D, this is apparently an unexplored possibility.

## Conclusion

While transient hypophosphatemia following parathyroidectomy in ESRD has been previously observed, this report highlights the possibility that this hypophosphatemia may persist if hypoparathyroidism persists long-term. Low PTH in patients on dialysis, such as (*a*) our patient reported here, (*b*) those in the literature,^2-4^ and (*c*) those receiving Cinacalcet^5-19^ or Velcalcetide^21^ do regularly lead to a decrease in serum Pi or overt hypophosphatemia. The long-term results with Cinacalcet indicate that this may not be transient, but a direct consequence of the decrease in PTH. The difference in degree may be related to the degree of hypoparathyroidism.
